# Editorial: Targeting Thyroid Hormone-Dependent Pathways in Proliferative and Degenerative Disorders

**DOI:** 10.3389/fmed.2022.944769

**Published:** 2022-06-08

**Authors:** Andrea Perra, Michelina Plateroti

**Affiliations:** ^1^Unit of Oncology and Molecular Pathology, Department of Biomedical Sciences, University of Cagliari, Cagliari, Italy; ^2^Université de Strasbourg, Inserm, IRFAC/UMR-S1113, FMTS, Strasbourg, France

**Keywords:** thyroid hormones, cell proliferation, T3 analogs, neurological disorders, liver cancer

Thyroid hormones (THs) are involved in the control of numerous cellular functions essential for proliferation, differentiation, and metabolism. Alterations of the molecular pathways controlled by THs have been recognized at the basis of numerous human pathologies, including metabolic diseases, tumors, and some neurodegenerative diseases. The possibility to treat these diseases by modulating the THs/thyroid hormone receptors (THs/THRs) axis is intriguing, in particular for pathologies for which, to date, no effective therapy exists. This is the case of hepatocellular carcinoma (HCC), the most frequent primary liver cancer (1).

To ameliorate the outcome of HCC, researchers recently focused their efforts on the possibility to target new molecular pathways involved in both cell proliferation, differentiation, metabolism, and cyto-protection. Deregulation of the THs/THRs axis has recently been characterized in human HCC and represents an early event in experimentally-induced liver carcinogenesis in rodents (2). Furthermore, intracellular hypothyroidism has been associated with non-alcoholic fatty liver disease (NAFLD), that in the next few years will become the main risk factor for chronic and neoplastic liver disease (3). In animal models of both chemically-induced and NAFLD-related HCC, the reactivation of the THs/THRs pathway by the administration of high doses of triiodothyronine (T3) led to the regression of the neoplastic lesions. It had been postulated that T3 was able to induce a differentiation program associated with a metabolic rewiring from glycolysis to oxidative phosphorylation (OXPHOS) (4). Together with its anti-tumorigenic potential, T3 has also been shown to reduce serum lipids (5) and amelioration of liver fibrosis (6). The possible mechanisms by which THs balance and THs/THRs activation influence the hepatocarcinogenic process and the potential utility of THs and their analogs for the treatment of HCC has been in-depth reviewed by Lin et al. In their review, Lin and colleagues, describe the major molecular pathways modulated by THs, starting from those controlling proliferation and differentiation. Surprisingly enough, despite its pro-mitogenic effects on normal hepatocytes, T3 demonstrates anti-tumoral effects mediated both by nuclear receptors and cell membrane receptors. Indeed, THs mediate both non-genomic actions through their interaction with the cell surface receptor integrin αvβ3, and genomic actions by modulating the transcriptional activity of nuclear THRs, which are ligand-dependent transcription factors. Moreover, THRs regulate cell proliferation and metabolism also directly regulating cytoplasmic signaling pathways. TH-regulated pathways have been described to be altered not only in human HCC biopsies, but also in preneoplastic hepatic lesions obtained from experimental animal models, clearly indicating their importance in hepatocarcinogenesis. The *in vivo* and *in vitro* demonstration of the antitumoral effects of T3 prompted the scientists to design and synthetize T3-analogs, selective for the THRβ isoform, devoid of the cardiovascular side-effects that limited its use.

Indeed, high doses of T3 required to induce the anti-steatotic and anti-tumoral effect, also cause severe systemic hyperthyroidism, a life-threatening condition. Since most of the harmful effects of T3, directed toward cardiovascular system, are mediated by the alpha-isoform of THRs (THRα), several new T3 analogs selective for the beta isoform of THRβ have been synthesized. Even if some of them entered clinical trials, they have been terminated because of the induction of adverse effects or because they failed to meet the clinical end points. Among these compounds, the first THRβ selective agonist demonstrating *in vivo* anti-tumoral effects on liver lesions was Sobetirome (GC-1) (7), a drug originally designed to treat hypercholesterolemia and mixed dyslipidemias. *In vivo* effects of GC-1 on both NAFLD and liver carcinogenesis led to the synthesis of other GC-1-based more liver-selective compounds. A new halogen-free THRβ selective molecule (TG68), derived from GC-1, demonstrated an anti-steatogenic activity *in vitro* and *in vivo*, in mice fed high fat diet (HFD) in the absence of systemic side effects (8). Furthermore, the administration of another new THRβ selective compound, Resmetirom, to patients with non-alcoholic steatohepatitis (NASH) led to a significant reduction in hepatic fat content (9).

The interest of clinicians and researchers for the development of novel selective analogs of T3 goes beyond their potential anti-tumoral actions on HCCs. In fact, TH/THRs pathway is also involved in several common human pathologies, such as dyslipidemias, metabolic, cardiovascular and neurodegenerative diseases. The correlation between THs and serum lipids concentration has been established long time ago. Recently, Wu et al. demonstrated an intriguing, although unexpected, association between the diagnosis of hyperthyroidism and abnormal blood lipids profile. In particular, the authors studied the correlation between THs levels, lipids concentration and anti-thyroid drugs (ATDs) in more than 13,000 hyperthyroid patients (in 13,667 patients). Their observations suggested that the abnormality in blood lipids was induced by the treatment of the anti-thyroid drugs 6-n-propyl-2-thiouracil (PTU) and methimazole (MMI). Moreover, the study suggested that hyperthyroid patients without hyperlipidemia undergoing standard treatment with anti-thyroid drugs showed significantly higher risk of developing hyperlipidemia during the follow-up.

Even more complex results were obtained by linking THs and cognitive performance. Indeed, whereas T4 levels positively correlate with better general cognition, in patients with mild cognitive impairment T3 levels inversely correlate cognitive performance. Starting from the evidence that subjects carrying the ε4 variant allele of the apolipoprotein E have an increased risk to develop mild cognitive impairment or dementia, Lee et al. investigated whether there was a correlation between the presence of this allele and THs levels. The findings of this study were really fascinating, as the authors demonstrated that higher levels of T3 could be protective in subjects with mild cognitive impairment carrying the ε4 allele. These results suggest that T3 supplementation could have a potential therapeutic and preventive in cognitive deterioration and dementia in APOE ε4 carriers. THs supplementation have been proposed also for other neuropsychological diseases, such as maniac and depressed phases of mood disorders and for neurodegenerative diseases such as multiple sclerosis (10). As discussed in the review by Saponaro et al., there is currently a clear evidence that modulation of THs/THRs pathways could represent an effective strategy for the treatment of metabolic and neurologic diseases. These findings have further increased the efforts applied in the development of TH analogs capable of uncoupling beneficial actions on liver and central nervous system from deleterious effects on the heart, muscle and bone. In the last decades, several AryloxyPhenyl based thyromimetics and Diphenylmethane based THRβ-selective agonists have been developed. Some of these molecules, like Sobetirome and its central nervous system selective prodrug Sob-AM2, demonstrated encouraging results on brain and spinal cord re-myelination.

Despite these encouraging new data, a more complete knowledge of the mechanisms through which THs and their analogs modulate cell functions will be fundamental, to design new therapeutic strategies for the treatment and prevention of some of the most common human diseases. In particular, the development of molecules that selectively activate specific THR isoforms has opened up the possibility of dissecting proliferative, metabolic, neuroprotective and antineoplastic effects, allowing, in the coming years, the development of new targeted therapies for regenerative medicine and cardiometabolic, neurodegenerative, and neoplastic pathologies.

## Author Contributions

All authors listed have made a substantial, direct, and intellectual contribution to the work and approved it for publication.

## Conflict of Interest

The authors declare that the research was conducted in the absence of any commercial or financial relationships that could be construed as a potential conflict of interest.

## Publisher's Note

All claims expressed in this article are solely those of the authors and do not necessarily represent those of their affiliated organizations, or those of the publisher, the editors and the reviewers. Any product that may be evaluated in this article, or claim that may be made by its manufacturer, is not guaranteed or endorsed by the publisher.
